# Effects of macrophages on the proliferation and cardiac differentiation of human induced pluripotent stem cells

**DOI:** 10.1186/s12964-022-00916-1

**Published:** 2022-07-18

**Authors:** Canling Long, Rui Guo, Ruijuan Han, Kang Li, Yanbing Wan, Jiqing Xu, Xiaoyu Gong, Yanqiu Zhao, Xinhuang Yao, Jia Liu

**Affiliations:** 1 Central Laboratory, The Second Affiliated Hospital, School of Medicine, The Chinese University of Hong Kong, Shenzhen & Longgang District People’s Hospital of Shenzhen, Shenzhen, 518172 Guangdong China; 2 Department of Cardiology, The Second Affiliated Hospital, School of Medicine, The Chinese University of Hong Kong, Shenzhen & Longgang District People’s Hospital of Shenzhen, Shenzhen, 518172 Guangdong China; 3 Cardiothoracic Surgery Department, The Second Affiliated Hospital, School of Medicine, The Chinese University of Hong Kong, Shenzhen & Longgang District People’s Hospital of Shenzhen, Shenzhen, 518172 Guangdong China

**Keywords:** Macrophage, iPSCs, Cardiac differentiation, Inflammation

## Abstract

**Background:**

Macrophage phenotypes switch from proinflammatory (M1) to anti-inflammatory (M2) following myocardial injury. Implanted stem cells (e.g., induced pluripotent stem cells (iPSCs)) for cardiomyogenesis will inevitably contact the inflammatory environment at the myocardial infarction site. To understand how the macrophages affect the behavior of iPSCs, therefore, improve the therapeutic efficacy, we generated three macrophage subtypes and assessed their effects on the proliferation, cardiac differentiation, and maturation of iPSCs.

**Methods:**

M0, M1, and M2 macrophages were polarized using cytokines, and their properties were confirmed by the expression of specific markers using reverse transcription-quantitative polymerase chain reaction (RT-qPCR) and immunofluorescence. The effects of macrophages on iPSCs were studied using Transwell co-culture models. The proliferative ability of iPSCs was investigated by cell counting and CCK-8 assays. The cardiac differentiation ability of iPSCs was determined by the cardiomyocyte (CM) yield. The maturation of CM was analyzed by the expression of cardiac-specific genes using RT-qPCR, the sarcomere organization using immunofluorescence, and the mitochondrial function using oxidative respiration analysis.

**Results:**

The data showed that the co-culture of iPSCs with M0, M1, or M2 macrophages significantly decreased iPSCs’ proliferative ability. M2 macrophages did not affect the CM yield during the cardiac differentiation of iPSCs. Still, they promoted the maturation of CM by improving sarcomeric structures, increasing contractile- and ion transport-associated gene expression, and enhancing mitochondrial respiration. M0 macrophages did not significantly affect the cardiomyogenesis ability of iPSCs during co-culture. In contrast, co-culture with M1 macrophages significantly reduced the cardiac differentiation and maturation of iPSCs.

**Conclusions:**

M1- or M2-polarized macrophages play critical roles in the proliferation, cardiac differentiation, and maturation of iPSCs, providing knowledge to improve the outcomes of stem cell regeneration therapy.

**Video abstract**

**Supplementary Information:**

The online version contains supplementary material available at 10.1186/s12964-022-00916-1.

## Introduction

Myocardial infarction (MI) remains the primary cause of mortality in patients with cardiovascular diseases worldwide [1, 2]. The massive loss of cardiomyocytes (CMs) during MI results in irreversible damage to the heart, eventually leading to heart failure [3]. One strategy to overcome the decreased cardiac performance post-MI is to replace dead tissue with functional CMs. Human induced pluripotent stem cell (iPSC)-derived CMs (iPSC-CMs) represent many aspects of human CMs, such as cardiac gene expression signatures, contractile function, electrophysiological properties, and metabolic characteristics, and have become a useful tool in cardiovascular studies, as well as a cell source for heart regeneration [4–6]. Many studies support the potential therapeutic benefits of iPSCs implanted in the injured myocardium, such as reducing the cardiac scar size and improving cardiac functions [7–10].

Macrophages are a key cell type involved in the inflammatory process post-MI [11, 12]. It is well recognized that following MI, the heart experiences monocyte invasion in the MI microenvironment [13]. M0 macrophages differentiated from monocytes are mature macrophages with a larger and more flattened morphology than monocytes. A switch from the M1 to M2 subtype is occurred during the healing process, suggesting the differential contribution of these macrophage subtypes to the post-MI microenvironment [14–18]. M1 macrophages involved in 1–3 days post-MI display a proinflammatory phenotype with increased proinflammatory cytokine secretion. In contrast, M2 macrophages secrete high levels of anti-inflammatory cytokines and promote angiogenesis and tissue remodeling on days 5–7 post-MI.

Once transplanted, iPSCs are surrounded by the inflammatory environment post-MI, which consists of inflammatory cells such as M1 and M2 macrophages. However, little is known about the effects of different macrophage subtypes on iPSC behavior. Here, we addressed these issues by studying the effect of exposing M0, M1, and M2 macrophages to iPSCs, in terms of proliferation, cardiac differentiation, and maturation, using co-culture models.

## Materials and methods

### Polarization of macrophages

THP-1 cells were cultured and polarized according to established protocols [19]. Briefly, THP-1 cells were cultured in RPMI 1640 medium supplemented with 10% fetal bovine serum (FBS). An amount of 1 × 10^6^ cells was plated in 6-well plates with 2 ml of medium containing 25 ng/ml PMA (catalog number: P8139; Sigma-Aldrich) for 2 days to acquire adherent cells (M0 macrophages). M0 macrophages were then treated for another 2 days with 20 ng/ml IFN-γ (PeproTech, catalog number: 300-02) and 100 ng/ml LPS (Sigma-Aldrich, catalog number: L2630) or with 20 ng/ml IL-4 (PeproTech, catalog number: 200–04) and 20 ng/ml IL-13 (PeproTech, catalog number: 200-13) to yield M1 or M2 polarized macrophages, respectively.

### Co-culture transwell system

In total, 8 × 10^4^ macrophages were seeded in a 0.4 μm pore insert of 24-well Transwell plates with 200 μl of PSCeasyII culture medium (Cellapy, catalog number: CA1014500) or differentiation medium (Cellapy, catalog number: CA2004500). Approximately 5 × 10^4^ iPSCs were cultured on Matrigel-coated (Cellapy, catalog number: CA3003100) wells with 800 μl of PSCeasyII culture medium for the proliferation study. After 6 days of cardiac differentiation, the cardiac mesoderm precursors were co-cultured with macrophages in the differentiation medium for cardiac maturation studies.

### Cell viability of iPSCs

After adding 10 μl of CCK8 solution (Beyotime, catalog number: C0038), the cells were incubated at 37 °C for 1 h. Optical density (OD) at 450 nm was recorded using a microplate reader (BioTek).

### Human iPSC culture

Human iPSCs derived from urine renal epithelial cells of a 37-year-old man were purchased from Cellapy Biological Technology Company (Beijing, China, catalog number: CA1002008). Human iPSCs were cultured as previously described [20, 21]. Briefly, iPSCs were cultured on Matrigel-coated plates with PSCeasyII culture medium (modified Essential 8 medium) at 37 °C in a humidified atmosphere of 5% CO_2_. The medium was refreshed every day, and the cells were routinely split 1:10, after reaching 70–80% confluence, onto pre-coated plates using dissociation medium (Cellapy, catalog number: CA3001500).

### Differentiation of iPSCs into CMs

Cardiac differentiation of iPSCs was performed using the CardioEasy Human Cardiomyocyte Differentiation Kit (Cellapy, catalog number: CA2004500). Briefly, cells were seeded at a confluence of 80–90% before differentiation. Then, the cardiac mesoderm precursors were induced using medium I for 2 days, subsequently changing to medium II for another 2 days. The medium was then changed to medium III and refreshed every 2 days. Contracting cells were observed at approximately day 8. On day 12, purification medium (Cellapy, catalog number: CA2005100) was used to metabolically select and purify CMs.

### Bright-field images

Bright-field images were captured using a Mshot MF53-N microscope with a 10 × PhL contrast filter lens.

### Immunofluorescence analysis

Immunostaining of cultured cells was carried out as previously described [22] using primary antibodies at a dilution of 1:200 in PBS containing 5% bovine serum albumin (Sigma-Aldrich, catalog number: A1933) as follows: rabbit anti-CCR7 (Beyotime, catalog number: AF1102), rabbit anti-CD36 (Beyotime, catalog number: AF6414), and rabbit anti-α-actinin (Proteintech, catalog number: 14221–1-AP). The secondary antibodies used were Alexa 488-conjugated goat anti-rabbit IgG (H + L) (Thermo Fisher Scientific, catalog number: A32731) and Alexa 568-coupled goat anti-rabbit IgG (H + L) (Thermo Fisher Scientific, catalog number: A11010). The secondary antibodies were diluted 1:400 in PBS. Nuclei were visualized using Hoechst 33,342 (10 µg/µl; Thermo Fisher Scientific, catalog number: H3570). Stained cells were imaged using a Zeiss LSM 900 confocal laser scanning microscope.

### Total RNA extraction and reverse transcription-quantitative polymerase chain reaction (RT-qPCR)

RT-qPCR was performed as previously described [23]. Total RNA was isolated using TRIzol reagent (Invitrogen, catalog number: 15596026), according to the manufacturer's protocol. RNA was reverse transcribed using the PrimeScript RT reagent kit (Takara, catalog number: RR037A). The expression levels of the genes of interest were determined by PCR using TB Green Premix Ex Taq (Takara, catalog number: RR420A) with primer pairs (Additional file 2: Table S1). PCR amplifications were performed using the QuantStudio 3 Real-Time PCR System (Thermo Fisher Scientific). Gene expression levels were normalized to those of the tyrosine 3-monooxygenase/tryptophan 5-monooxygenase activation protein zeta (*YWHAZ*) or glyceraldehyde-3-phosphate dehydrogenase (*GAPDH*) housekeeping genes. Quantitative data were analyzed based on the 2^−ΔΔCT^ method.

### Oxidative respiration analysis

A high-resolution Oxygraph-2 k respirometer (Oroboros) was used in this study. Oxidative respiration was performed as previously described [24, 25]. Briefly, cells were trypsinized, washed with PBS, resuspended at a density of 2 × 10^4^ cells/ml in DMEM supplemented with 10% FBS and transferred to the chamber of the Oxygraph-2 k instrument. Total oxygen consumption was monitored and the following drugs were injected into the medium as follows: oligomycin (3.5 μM), FCCP (4 μM), antimycin A (2 μM), and rotenone (2 μM) (all from Sigma-Aldrich).

### Statistical analysis

Data are presented as the mean ± standard error of the mean (SEM). Statistical analysis was performed using GraphPad Prism 8 (GraphPad Software, San Diego, CA, USA) using one-way ANOVA with Bonferroni's test for multiple comparisons or a Student's *t*-test for comparisons between two groups. All experiments were independently replicated at least three times. Statistical significance was set at P < 0.05.

## Results

### Polarization and characterization of macrophages

**Fig. 1:**
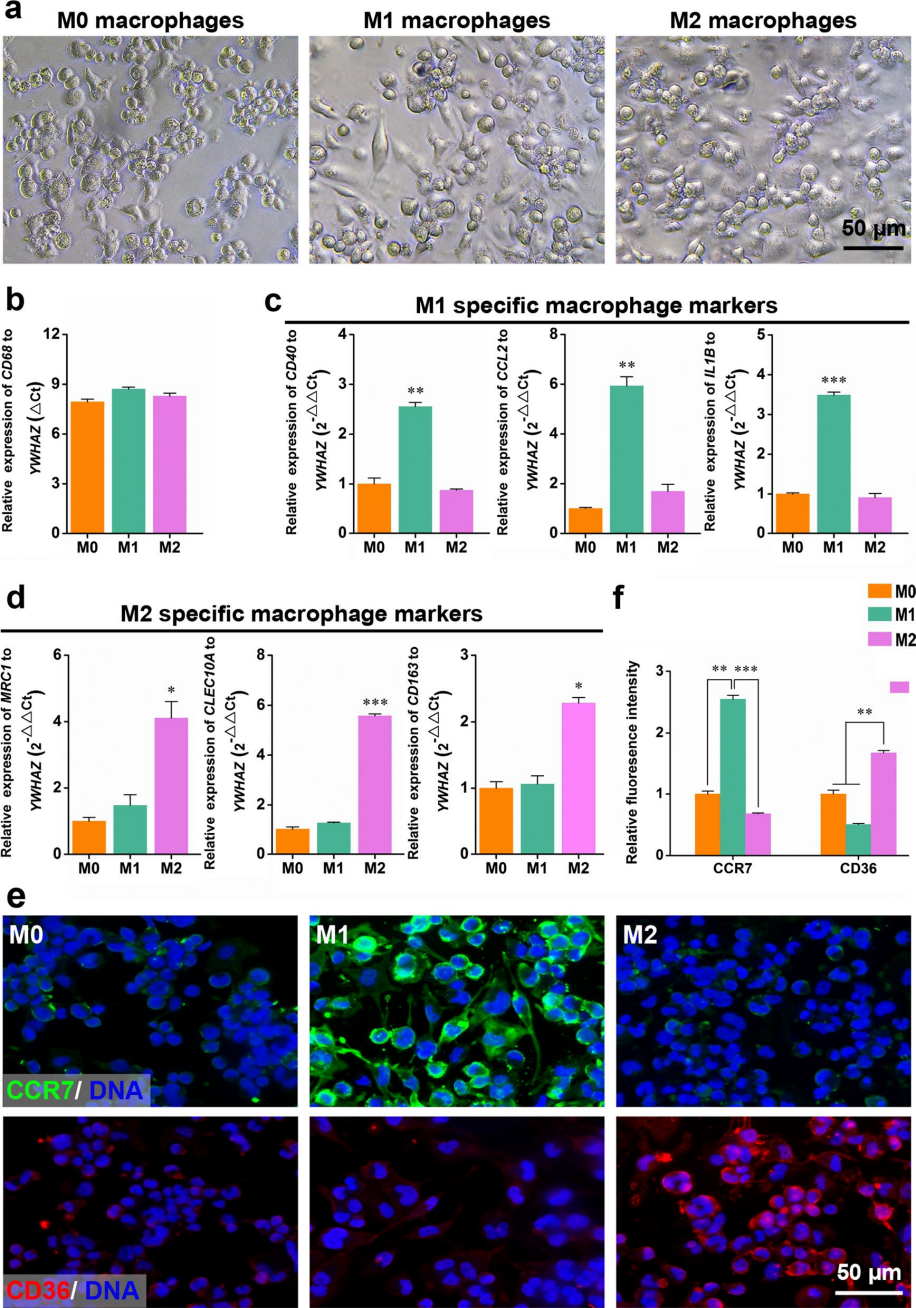
Characterization of polarized macrophages. **a** Bright-field images showing the morphological appearance of M0, M1, and M2 macrophages. **b**–**d** RT-qPCR analysis of the specific macrophage markers expression, such as general marker for macrophages CD68 (**b**), M1 markers CD40, IL1b and CCL2 (**c**), M2 markers MRC1, CLEC10a, and CD163 (**d**). **e** Fluorescence image of M0, M1, and M2 macrophages immunostained for M1-marker CCR7 (green) and M2-marker CD36 (red). Cell nuclei were counterstained with Hoechst 33,342 (blue). **f** The quantification of relative fluorescence intensity of CCR7and CD36 by ImageJ. mRNA levels are shown relative to those of M0 macrophage except for CD68, which were set at 1. Data are presented as mean ± SD, N = 3 individual preparations. Statistics were done by one-way ANOVA test with Bonferroni correction. *P < 0.05, **P < 0.01, ***P < 0.001

THP-1 monocytes adhered in response to PMA to generate M0 macrophages. M0 macrophages were further polarized into M1 and M2 macrophages upon stimulation with LPS and IFN-γ or IL4 and IL13, respectively (Fig. 1a). The three macrophage subtypes exhibited high expression of *CD68*, which is a general macrophage marker (Fig. 1b), compared to levels of the internal control gene *YWHAZ*. The significant upregulation of *CD40*, *IL1B*, and *CCL2* was observed in M1 macrophages compared to levels in M0 and M2 macrophages (Fig. 1c). In contrast, significantly higher *MRC1*, *CLEC10A*, and *CD163* expression was observed in M2 macrophages relative to that in M0 and M1 macrophages (Fig. 1d).

Immunofluorescence for macrophage subtype markers was used to further assess the M0, M1, and M2 macrophage populations (Fig. 1e). M0 macrophages showed slight positive staining for the M1 marker CCR7 and M2 marker CD36. M1 macrophages showed positive staining for CCR7 and negative staining for CD36. In contrast, M2 macrophages were positively stained for CD36 and less positively stained for CCR7. Consistently, quantifying the fluorescence intensity from specific macrophage markers showed significant differences among the three macrophage populations (Fig. 1f). The expression of CCR7 was significantly higher in M1 macrophages than in M0 and M2 macrophages. In contrast, CD36 was more highly expressed in M2 macrophages than in M0 or M1 macrophages.

### M0, M1, and M2 macrophages suppress the cell viability of iPSCs.

**Fig. 2:**
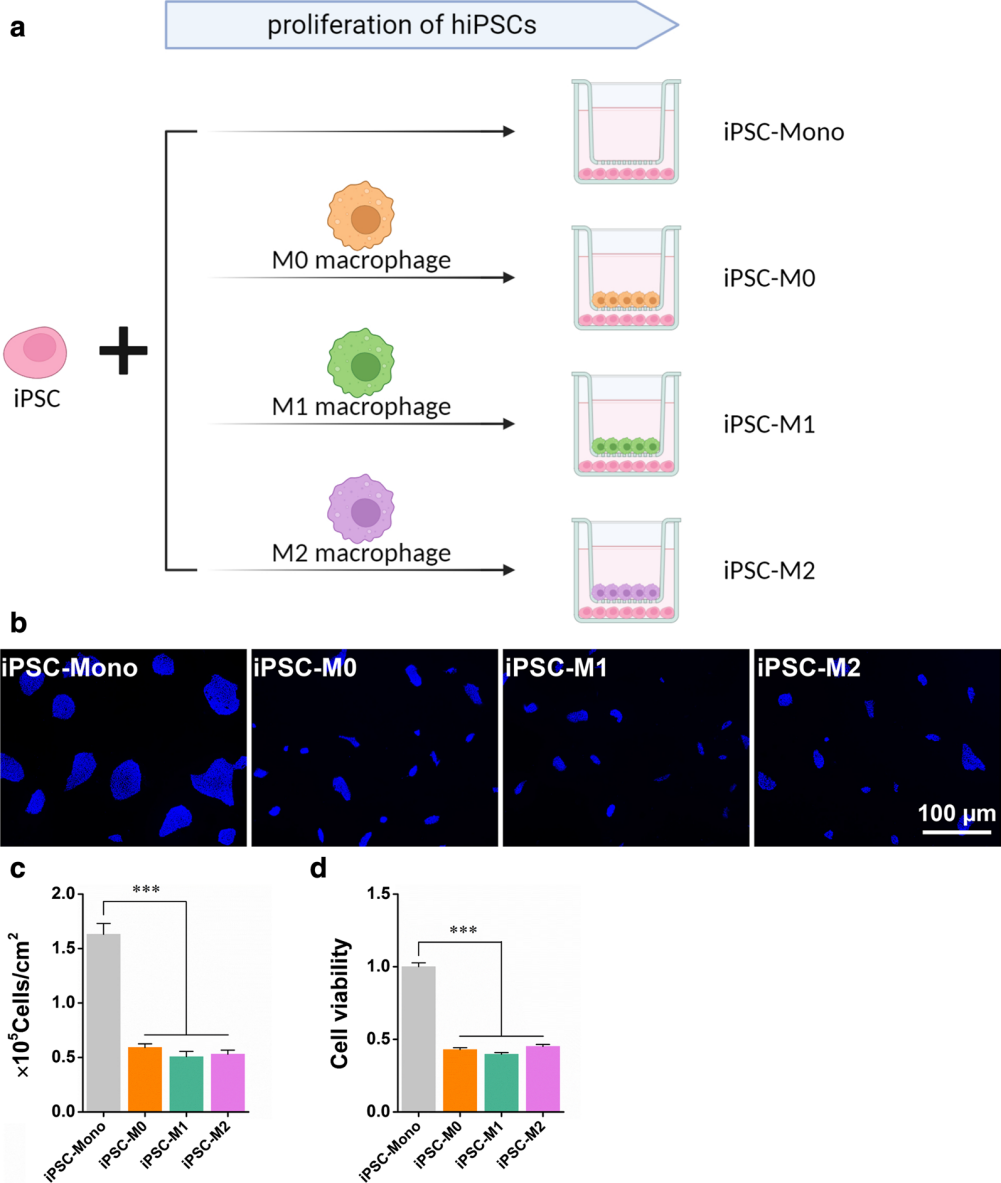
Cell proliferation of iPSCs co-cultured with M0, M1, or M2 macrophages. **a** Scheme showing the set-up of iPSCs proliferation in a monoculture or co-cultured with M0, M1, and M2 macrophages using a transwell system. **b** Fluorescence image of the cell nuclei of iPSCs monoculture or co-cultured for 3 days with M0, M1, and M2 macrophages stained with Hoechst 33,342 (blue). **c** Quantification of cell nuclei in iPSCs cultures with indicated conditions. **d** Cell viability analysis of iPSCs cultures with indicated conditions performed by CCK8. iPSC-Mono, iPSCs monoculture; iPSC-M0, iPSCs co-cultured with M0; iPSC-M1, iPSCs co-cultured with M1; iPSC-M2, iPSCs co-cultured with M2. Data are presented as mean ± SEM, N = 3 individual preparations. Statistics were done by one-way ANOVA test with Bonferroni correction. ***P < 0.001

To investigate whether macrophages affect the viability and proliferation of iPSCs, M0, M1, and M2 macrophages were co-cultured with iPSCs using a Transwell system (Fig. 2a). After 3 days of co-culture, all three macrophage subtypes significantly inhibited the proliferative capacity of iPSCs, as shown by the smaller cell cluster sizes and decreased cell numbers in iPSC-M0, iPSC-M1, and iPSC-M2 groups compared with those in the iPSC-Mono group (Fig. 2b, c). Consistently, the viability of iPSCs in iPSC-M0, iPSC-M1, and iPSC-M2 groups was lower than that in the iPSC-Mono group, indicating that M0, M1, and M2 macrophages suppress the viability of iPSCs (Fig. 2d).

### Effects of macrophages on cardiomyogenic differentiation capacity of iPSCs

**Fig. 3:**
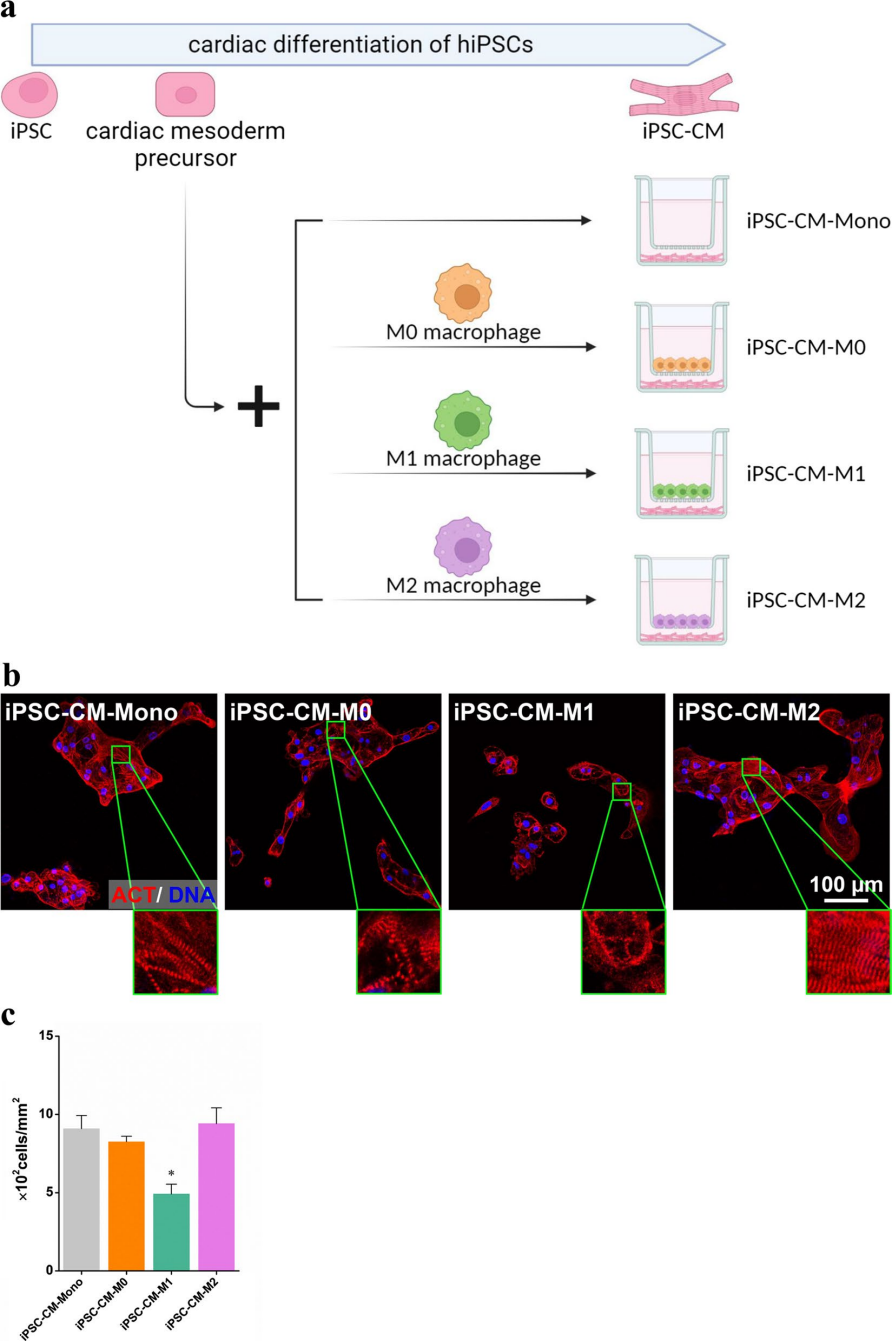
Cardiac differentiation capacity of iPSCs co-cultured with M0, M1, and M2 macrophages. **a** Scheme showing the set-up of iPSCs cardiomyogenic differentiation in a monoculture or co-cultured with M0, M1, and M2 macrophages using a transwell system. Cardiac mesoderm precursors were first derived from iPSCs. Then the cardiac mesoderm precursors continued cardiomyogenic differentiation in a monoculture or co-cultured with M0, M1, and M2 macrophages using a transwell system. **b** Fluorescence image of iPSC-CM-Mono, iPSC-CM-M0, iPSC-CM-M1, and iPSC-CM-M2 stained for sarcomeric α-actinin (red). Cell nuclei were counterstained with Hoechst 33,342 (blue). **c** Quantification of cell nuclei in cultures with indicated conditions by ImageJ. iPSC-CM, iPSCs derived CM; iPSC-CM-Mono, iPSC derived CM in monoculture; iPSC-CM-M0, iPSCs derived CM in a co-culture with M0; iPSC-CM-M1, iPSCs derived CM in a co-culture with M1; iPSC-CM-M2, iPSCs derived CM in a co-culture with M2

To evaluate the influence of macrophages on the cardiomyogenic capacity, iPSCs were subjected to cardiomyogenic differentiation in the presence of M0, M1, and M2 macrophages (Fig. 3a). iPSCs were first differentiated into cardiac mesoderm precursors to initiate cardiomyogenic differentiation and subsequently differentiated into CMs with or without exposure to macrophages. After CM purification at the end of the differentiation period, almost all cells were stained positive for the cardiac sarcomeric protein α-actinin in all four culture conditions, that is iPSC-CM-Mono, iPSC-CM-M0, iPSC-CM-M1, and iPSC-CM-M2 groups. iPSC-CM-M0 and iPSC-CM-M2 groups achieved similar CM yields and possessed highly organized sarcomeres, as with that observed in the iPSC-CM-Mono group. However, the cell yield was significantly lower in the iPSC-CM-M1 culture than in the iPSC-CM-Mono culture (Fig. 3b, c). These findings suggest that M1 macrophages inhibit the cardiomyogenic differentiation capacity of iPSCs in terms of the CM yield.

### Effects of macrophages on the myofibrillogenesis of hiPSC-CMs

**Fig. 4:**
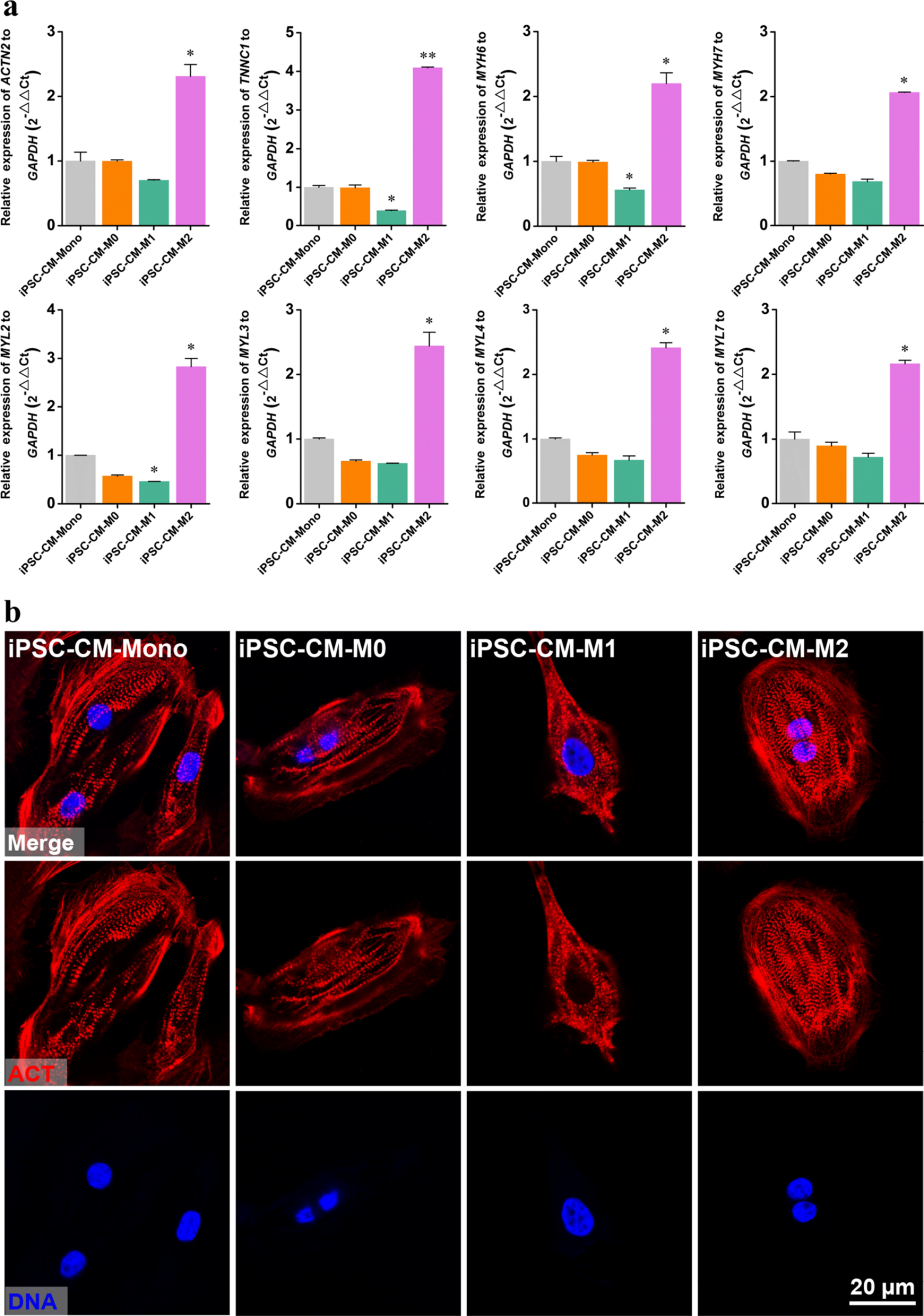
Structural maturation of iPSC-CMs co-cultured with M0, M1, or M2 macrophages. **a** RT-qPCR analysis of the cardiac sarcomeric genes expression. **b** Fluorescence image of cardiac differentiated iPSC cultures co-cultured with M0, M1, or M2 macrophages immunostained for α-actinin (red). Cell nuclei were counterstained with Hoechst 33,342 (blue). mRNA levels are shown relative to those of M0 macrophage, which were set at 1. Data are presented as mean ± SEM, N = 3 individual preparations. Statistics were done by one-way ANOVA test with Bonferroni correction. *P < 0.05

To determine the effects of macrophages on the myofibrillogenesis of iPSC-CMs, we examined sarcomeres in iPSC-CMs co-cultured with different macrophage subtypes. RT-qPCR analysis of iPSC-CM-Mono culture versus iPSC-CM-M2 culture revealed enhanced CM structural maturation in the latter, with upregulation of the crucial cardiac sarcomeric genes *ACTN2*, *TNNC1*, *MYH6*, *MYH7*, *MYL2*, *MYL3*, *MYL4*, and *MYL7*, which are essential for sarcomere assembly and function in the mammalian heart (Fig. 4a). In contrast, iPSC-CM-M1 cultures showed decreased cardiac sarcomeric gene expression compared to that in the iPSC-CM-Mono group. A similar trend in sarcomeric gene expression was observed for iPSC-CM-M0 and iPSC-CM-Mono groups (Fig. 4a).

Furthermore, immunostaining for α-actinin in iPSC-CMs confirmed improved sarcomere organization in the iPSC-CM-M2 culture compared to that in the iPSC-CM-Mono group (Fig. 4b). In the iPSC-CM-M1 group, α-actinin formed a punctate staining pattern reminiscent of immature myofibrils, consistent with their decreased sarcomeric gene expression (Fig. 4b). These data revealed that M2 macrophages improved, whereas M1 macrophages inhibited, the structural maturation of iPSC-CMs.

### Effects of macrophages on ion transport-related gene expression in iPSC-CMs

**Fig. 5:**
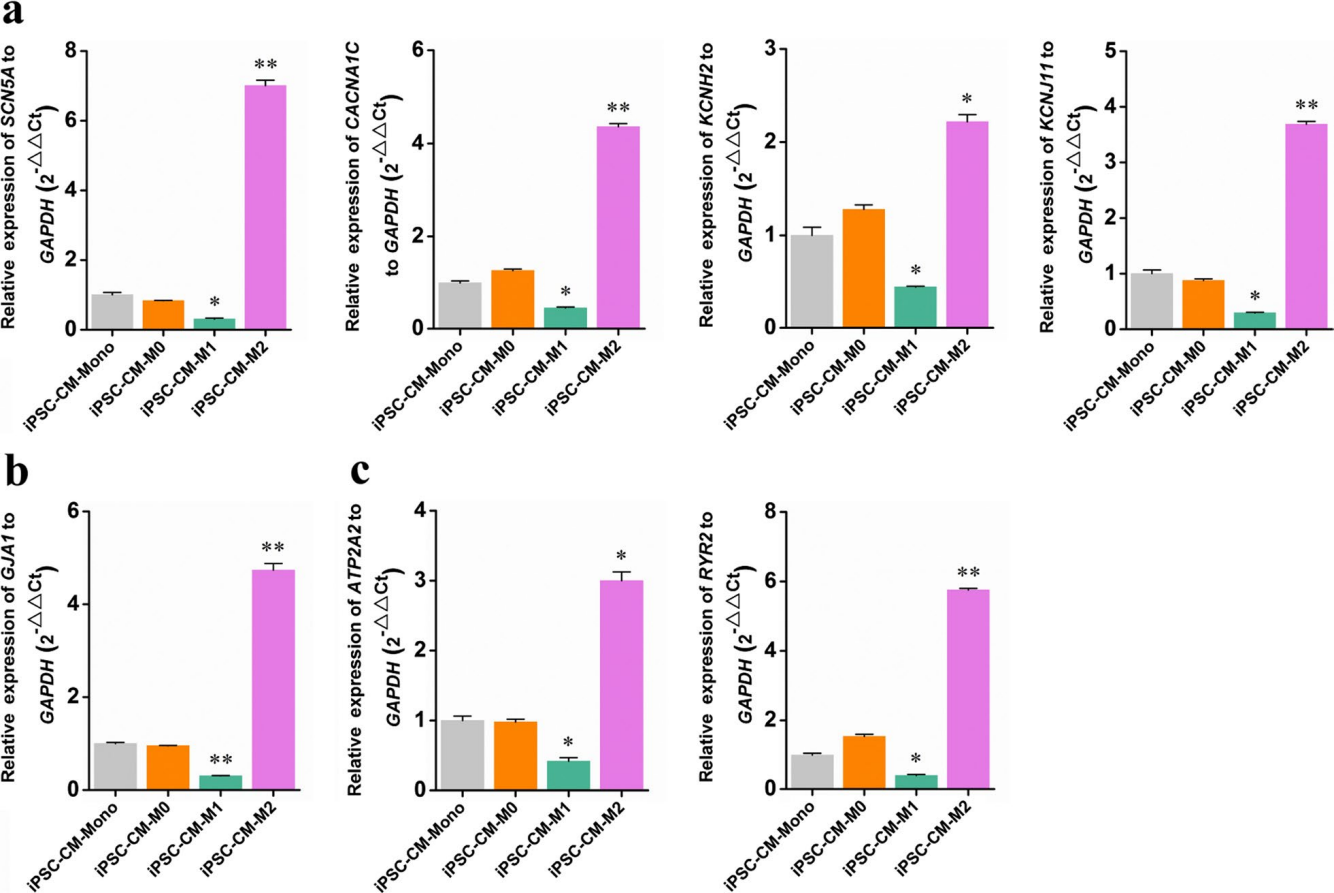
Analysis of the cardiac ion transport-related gene expression in hiPSC-CMs co-cultured with different macrophages. The mRNA levels of ion channel genes (**a**), gap junction protein gene (**b**), and cardiac Ca^2+^-handling protein genes (**c**) were tested by RT-qPCR. The mRNA levels are shown relative to those of the M0 macrophage, which were set at 1. Data are presented as mean ± SEM, N = 3 individual preparations. Statistics were done by one-way ANOVA test with Bonferroni correction. *P < 0.05, **P < 0.01

To determine whether structural maturation was accompanied by electrical maturation, we compared the levels of ion transport-related gene expression in iPSC-CMs co-cultured with different macrophage types. RT-qPCR targeting genes encoding cardiac ion channels (*SCN5A*/Nav1.5, *CACNA1C*/Cav1.2, *KCNH2*/Kv11.1, and *KCNJ11*/Kir6.2), a gap junction protein (*GJA1*/Cx43), and cardiac Ca^2+^-handling proteins (*RYR2* and *ATP2A2*/Serca2) showed an increase in mRNA levels in iPSC-CM-M2 cultures compared to those in iPSC-CM-Mono cultures (Fig. 5a–c). The iPSC-CM-M0 group expressed approximately equal levels of ion transport-related genes relative to those in the iPSC-CM-Mono group (Fig. 5a–c). However, ion transport-related gene expression was significantly decreased in iPSC-CM-M1 cultures (Fig. 5a–c). These findings suggested that M2 macrophages increased, whereas M1 macrophages suppressed, the expression of ion transport-related genes.

### Effects of macrophages on metabolic maturation of iPSC-CMs

**Fig. 6:**
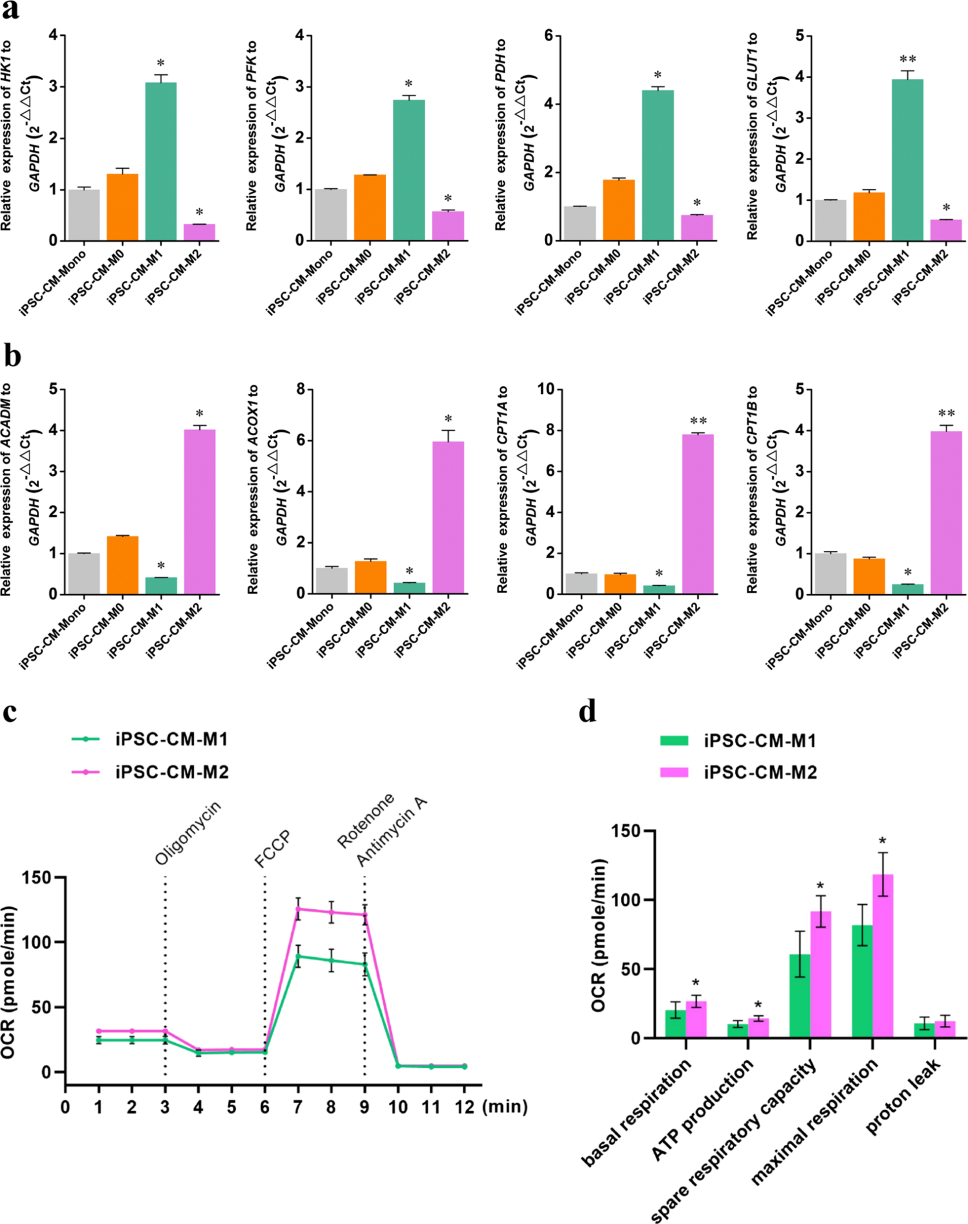
Metabolic maturation of hiPSC-CMs. **a** and **b** RT-qPCR analysis of the glycolysis-associated genes (**a**) and fatty acid β-oxidation genes expression (**b**). **c** Traces and **d** quantification graph for OCR measured by Seahorse respirometry in hiPSC-CMs co-cultured with M0, M1, or M2 macrophages. The mRNA levels are shown relative to those of the M0 macrophage, which were set at 1. Data are presented as mean ± SEM, N = 3 individual preparations. Statistics were done by one-way ANOVA test with Bonferroni correction. *P < 0.05, **P < 0.01, ***P < 0.001. OCR, oxygen consumption rate

To determine whether structural and electrical maturation was accompanied by enhanced metabolism, we first compared metabolic gene signatures among the four cultures using RT-qPCR analysis. RT-qPCR data showed a reduction in glycolysis-associated genes (*HK1*, *PFK*, *PDH*, and *GLUT1*) and an increase in fatty acid β-oxidation-associated genes (*ACADM*, *ACOX1*, *CPT1A*, and *CPT1B*) in iPSC-CM-M2 cultures compared to those in iPSC-CM-Mono cultures (Fig. 6a, b). In contrast, iPSC-CM-M1 cultures had a greater preference for glycolysis over mitochondrial respiration, as shown by the higher expression of glycolysis-associated genes and lower expression of fatty acid β-oxidation-associated genes compared to levels in the iPSC-CM-Mono group (Fig. 6a, b).

Mitochondrial respiration of M1 and M2 macrophages was measured using an Oxygraph-2 k respirometer. The mitochondrial respiration capacity of iPSC-CM-M2 cultures was significantly higher than that of iPSC-CM-M1 cultures (Fig. 6c, d). The enhanced mitochondrial respiratory capacity of the iPSC-CM-M2 group compared to that in the iPSC-CM-M1 group indicated that M2 macrophages enhanced the metabolic maturation of iPSC-CMs compared to that with M1 macrophages.

## Discussion

Myocardial infarction remains the leading cause of death worldwide. Many studies have shown that implanted iPSCs can diminish the cardiac scar size and improve functional recovery of the injured myocardium [26, 27]. A better understanding of how the surrounding inflammatory environment influences iPSC survival and cardiac differentiation capability is required to maximize therapeutic efficacy. However, previous studies have generally focused on the effects of macrophages on the existed cardiac fibroblasts and cardiomyocytes [28–30], mostly without considering the role of M1/M2 macrophages in the survival and cardiac differentiation of iPSCs. Therefore, in the present study, we differentiated and characterized three macrophage subtypes and assessed their effects on iPSC behavior. All three macrophage subtypes suppressed iPSC proliferation. M2 macrophages promoted the cardiac differentiation ability of co-cultured iPSCs in terms of sarcomeric structures, contractile and electrophysiological gene expression, and mitochondrial respiration. In contrast, M1 macrophages displayed the opposite effects compared to those of M2 macrophages.

Before the co-culture process, we evaluated the influence of the cardiac differentiation medium on the properties of macrophages. As shown in Additional files 1 and 3: Fig. S1a, the expression of *CD68*, a macrophage marker, was not significantly different between M0 macrophages cultured in a macrophage growth medium and those cultured in a cardiomyogenic differentiation medium. The same was observed for M1 and M2 macrophages regarding the expression of their specific markers (*IL1B*, *CD40*, *CCL2*, *MRC1*, *CLEC10A*, and *CD163*). Consistently, immunostaining for macrophage markers in macrophages cultured with a cardiomyogenic differentiation medium showed the same pattern as that with macrophage growth medium (Additional files 1 and 3: Fig. S1b and Fig. 1e). Taken together, these data indicated that the cardiac differentiation medium had no significant effect on the properties of M0, M1, and M2 macrophages. Therefore, complete cardiac differentiation medium was used to culture macrophages and generate iPSC-CMs over the co-culture period.

The CM yield in the iPSC-CM-M2 group was slightly higher than that in the iPSC-CM-Mono group (Fig. 3b, c). This phenomenon might result from the effects of M2 macrophages on BMP proteins expression in the co-cultured cells. Previous studies have proven that BMP proteins, especially BMP2, are key players in cardiogenesis [31–33]. Although the levels of BMP-2 secretion among M0, M1, and M2 macrophages were not significantly different [34], studies have shown that M2 macrophages have the ability to enhance autologous BMP-2 production in co-cultured cells [35, 36]. However, the role of BMP-2 in promoting the cardiac differentiation of iPSCs co-cultured with M2 macrophages requires further investigation.

Immunostaining for α-actinin in the culture after CM purification at the end of the differentiation period revealed that iPSC-CM-M2 cultures possessed highly organized sarcomeres, even better than those in the iPSC-CM-Mono group. In contrast, in the iPSC-CM-M1 group, α-actinin formed a punctate staining pattern, reminiscent of immature myofibrils (Fig. 4b). This is particularly relevant to the high expression levels of genes encoding cardiac sarcomeric and Ca^2+^-handling proteins in iPSC-CM-M2 cultures and the low expression levels in iPSC-CM-M1 cultures (Fig. 4a). This finding is consistent with a previous study, which indicated that M2 macrophages enhanced, whereas M1 macrophages reduced, the calcium-handling function of CMs [29]. Their study showed that the conditioned medium from M1 macrophages significantly decreased cardiac troponin T (*cTnT*) and sarcoplasmic/endoplasmic reticulum calcium ATPase (*Serca2/Atp2a2*) gene expression. It was also demonstrated that the co-culture of M2 macrophages significantly enhanced Ca^2+^ release in CMs [29, 37]. Several studies have investigated the role of macrophages on CM viability. One examined the effects of proinflammatory macrophages on the survival of rat H9c2 cells. They found that exposure to proinflammatory macrophages led to myocyte apoptosis via mitochondrial damage [38]. Another study reported that the addition of M1 conditioned medium decreased the proliferation ability of CMs compared to that of anti-inflammatory macrophages [39]. In addition, the suppression of macrophage infiltration or proinflammatory cytokine production facilitates cardiac healing and improves cardiac function recovery [40].

Different phenotypic and functional macrophage subtypes are along with dramatic metabolic differences. M1 macrophages exhibit increased glycolysis accompanied by decreased oxygen consumption [41, 42]. M1 macrophages rely on glycolysis to produce ATP, whereas their TCA cycle has two brakes [43, 44], thus reducing oxidative phosphorylation and fatty acid oxidation [43, 45, 46]. In contrast, the metabolic characteristics of M2 macrophages include intensive oxidative phosphorylation and fatty acid oxidation [43]. The TCA cycle of M2 macrophages is intact and meets their ATP requirements by providing substrates for the electron transport chain [43]. Some metabolic products function as mediators of key signaling pathways [47], affecting not only host cells but also adjacent cells. Thus, metabolic products generated from different macrophage subtypes might lead to significant differences in the metabolic signatures of co-cultured iPSC-CMs (Fig. 6).

Interestingly, we found that M2 macrophages enhanced the cardiac differentiation and maturation of iPSCs, whereas both M1 and M2 macrophages inhibited the proliferation of iPSCs. Future studies are required to fully understand the regulatory mechanisms of macrophages with respect to the behavior of iPSCs, specifically regarding mitochondrial function.

## Conclusions

In this study, we systematically investigated the influence of different macrophage subtypes on the proliferation, cardiac differentiation, and maturation of human iPSCs. We found that all three types of macrophages significantly reduced iPSC proliferation. Furthermore, we demonstrated that M2 macrophages had a beneficial effect on iPSC cardiomyogenesis by promoting their maturation in terms of the sarcomere structure, electrophysiological-associated gene expression, and mitochondrial functions. This enhanced effect was not observed when the cells were co-cultured with M0 macrophages. M1 macrophages, however, inhibited the cardiac differentiation and maturation of iPSCs. Our findings not only elucidated the critical role of macrophages in the proliferation and cardiomyogenesis of iPSCs but also provided essential guidance for the implantation of iPSCs into the infarcted heart for heart regeneration therapy.

## Supplementary Information

**Additional file 1.** Supplemental Figure Legends.

**Additional file 2.** Supplemental Table.

**Additional file 3.** Supplemental Figure.

## Data Availability

All data are available from the corresponding author on reasonable request.

## Abbreviations

iPSC: Induced pluripotent stem cell
CM: Cardiomyocyte
iPSC-CM: Induced pluripotent stem cell derived cardiomyocyte
MI: Myocardial infarction
PMA: Phorbol 12-tetradecanoate 13-acetate
LPS: Lithium phosphorus sulfide
IFN-γ: Interferon-gamma
IL4: Interleukin4
IL13: Interleukin13
YWHAZ: Tryptophan 5-monooxygenase activation protein zeta
IL1B: Interleukin 1 beta
CCL2: C–C motif chemokine ligand 2
MRC1: Mannose receptor C-type 1
CLEC10A: C-type lectin domain containing 10A
CCR7: C–C motif chemokine receptor 7
OD: Optical density
RT-qPCR: Reverse transcription-quantitative polymerase chain reaction
GAPDH: Glyceraldehyde-3-phosphate dehydrogenase
FBS: Fetal bovine serum
FCCP: Carbonyl cyanide p-trifluoromethoxyphenylhydrazone
cTnT: Cardiac troponin T
iPSC-M0: IPSCs co-culture with M0
iPSC-M1: IPSCs co-culture with M1
iPSC-M2: IPSCs co-culture with M2
iPSC-Mono: IPSCs monoculture
iPSC-CM-Mono: IPSC derived CM in monoculture
iPSC-CM-M0: IPSCs derived CM in a co-culture with M0
iPSC-CM-M1: IPSCs derived CM in a co-culture with M1
iPSC-CM-M2: IPSCs derived CM in a co-culture with M2
ACTN2: Actinin alpha 2
TNNC1: Troponin C1
MYH6: Myosin heavy chain 6
MYH7: Myosin heavy chain 7
MYL2: Myosin light chain 2
MYL3: Myosin light chain 3
MYL4: Myosin light chain 4
MYL7: Myosin light chain 7
SCN5A: Sodium voltage-gated channel alpha subunit 5
Nav1.5: Sodium channel protein 1.5
CACNA1C: Calcium voltage-gated channel subunit alpha1 C
Cav1.2: Calcium channel protein 1.2
KCNH2: Potassium voltage-gated channel subfamily H member 2
Kv11.1: Potassium channel protein 11.1
KCNJ11: Potassium inwardly rectifying channel subfamily J member 11
Kir6.2: ATP-sensitive potassium channel subunit 6.2
GJA1: Gap junction protein alpha 1
Cx43: Connexin 43
RYR2: Ryanodine receptor 2
ATP2A2: ATPase sarcoplasmic/endoplasmic reticulum Ca^2+^ transporting 2
Serca2: Sarcoplasmic/endoplasmic reticulum calcium ATPase 2
HK1: Hexokinase 1
PFK: 6-Phosphofructo-2-kinase/fructose-2,6-biphosphatase 3
PDH: Pyruvate dehydrogenase phosphatase catalytic subunit 1
GLUT1: Solute carrier family 2 member 1
ACADM: Acyl-CoA dehydrogenase medium chain
ACOX1: Acyl-CoA oxidase 1
CPT1A: Carnitine palmitoyltransferase 1A
CPT1B: Carnitine palmitoyltransferase 1B
