# Supplementary material for: Effects of macrophages on the proliferation and cardiac differentiation of human induced pluripotent stem cells
Source: Cell Commun Signal. 2022 Jul 18;20:108. doi: 10.1186/s12964-022-00916-1 (PMC9290307; doi:10.1186/s12964-022-00916-1)
Supplement: Supplementary file 2 — Additional file 1. Supplemental Figure Legends. [file 12964_2022_916_MOESM2_ESM.docx]

**Supplemental figure legends**

**Figure S1.** **The effects of cardiac differentiation medium on macrophages.**

(a) RT-qPCR analysis of the expression of the specific marker in macrophages with or without the treatment of cardiac differentiation medium (CDM). (b) Fluorescence image of M0, M1 and M2 macrophages with the cardiac differentiation medium treatment immunostained for M1-marker CCR7 (green) and M2-marker CD36 (red). Cell nuclei were counterstained with Hoechst 33342 (blue). mRNA levels are shown relative to those of M0 macrophage, which were set at 1. Data are presented as mean±SEM, N=3 individual preparations. Statistics were done by one-way ANOVA test with Bonferroni correction.
