# Supplementary material for: Effects of macrophages on the proliferation and cardiac differentiation of human induced pluripotent stem cells
Source: Cell Commun Signal. 2022 Jul 18;20:108. doi: 10.1186/s12964-022-00916-1 (PMC9290307; doi:10.1186/s12964-022-00916-1)
Supplement: Supplementary file 3 — Additional file 2. Supplemental Table. [file 12964_2022_916_MOESM3_ESM.docx]

**Supplemental table**

[**Table**](http://www.nature.com/nbt/journal/v32/n10/full/nbt.3002.html#supplementary-information) **1. Primer pairs used for gene expression analysis**

| **Gene** | **Forward primer** | **Reverse primer** |
| --- | --- | --- |
| *CD68* | CTACTGGCAGAGAGCACTGG | CCGCCATGTAGCTCAGGTAG |
| *CD40* | GGCAGGCACAAACAAGACTG | TGGCTTCTTGGCCACCTTTT |
| *CCL2* | GATCTCAGTGCAGAGGCTCG | TTTGCTTGTCCAGGTGGTCC |
| *IL1B* | AGCCATGGCAGAAGTACCTG | TGAAGCCCTTGCTGTAGTGG |
| *MRC1* | GCAGAAGGAGTAACCCACCC | TGGCAAATGAAGGCGTTTGG |
| *CLEC10A* | GCCAGGTGGCTACTCTCAAC | GCTTCCAATCTCCCAGTGCT |
| *CD163* | CTTGGGACTTGGACGATGCT | GGTATCTTAAAGGCTCACTGGGT |
| *YWHAZ* | CTCCCGTTTCCGAGCCATAA | AAGATGACCTACGGGCTCCT |
| *ACTN2* | CTTCTACCACGCTTTTGCGG | CGCTCCAGTCTCCGAATCTC |
| *TNNC1* | GGCCGCATCGACTATGATGA | CAGGACTCAGCTGGAGTTGG |
| *MYH6* | CCAGAGCTTGCTGAAGGACA | TTGGCAAGAGTGAGGTTCCC |
| *MYH7* | GTAGACACACTTGAGTAGCCCA | ACAGAAGAGGCCCGAGTAGG |
| *MYL2* | AGGCGGAGAGGTTTTCCAAG | GGACCACTCTGCAAAGACGA |
| *MYL3* | TACTTACAGCCCCCAATGGC | TCTTGCCCAGCCATCAACTT |
| *MYL4* | TGTGAGGCTTATCAACTGCTCA | TTGCCAATTGGTGGAAACCC |
| *MYL7* | AAGCCATCCTGAGTGCCTTC | AACATCTGCTCCACCTCAGC |
| *SCN5A* | CAGGAATCCCAGCCTGTGTC | CCACCCTCATGCCCTCAAAT |
| *CACNA1C* | CCTCTGCAGAAACAGCTCCT | CTTTCGCGCAAGATTCGAGG |
| *KCNH2* | TGGACACCATCATCCGCAAG | ATGGCTGTCACTTCGTCCAG |
| *KCNJ11* | GAAGAGTCTGGTGGGGAGTT | GATCATGCTCTTGCGGAGGT |
| *GJA1* | ACTAGCCATTGTGGACCAGC | CCATACACCCCCAGTGAACC |
| *ATP2A2* | CCTTGGATTTCCCGAGGCTT | CCAGTATTGCAGGTTCCAGGT |
| *RYR2* | TGAATTGCCAGCGTTGTGTG | ACTGATCACAGGTGGCTGAA |
| *HK1* | CGCAGCTCCTGGCCTATTA | CTTCCACTCCGCTCGCTTTA |
| *PFK* | GATGCCCTTCAGGAAAGCCT | GAACACTTTTGTGGGGACGC |
| *PDH* | GTATTCCCTACTCCCTGCCACG | TGGGATTCCAATTCGTCTGGG |
| *GLUT1* | TGAGCATCGTGGCCATCTTT | AGGCATGGAACCATTCAGGG |
| *ACADM* | GGCCGTGACCCGTGTATTAT | CTGCAGCATCGCCCGAA |
| *ACOX1* | AAGCTGGAGACTCCCTCGAT | TCCTGGCAAAGGCTTATGGG |
| *CPT1A* | CATACGAGGCCTCCATGACC | TTTCCAGCCCAGCACATGAA |
| *CPT1B* | ACTCCTGGAAGAAACGCCTG | CACAGACTCTAGGTAAGCCCAG |
| *GAPDH* | AATGGGCAGCCGTTAGGAAA | GCGCCCAATACGACCAAATC |
