# Supplementary figures and images for: Effects of macrophages on the proliferation and cardiac differentiation of human induced pluripotent stem cells
Source: Cell Commun Signal. 2022 Jul 18;20:108. doi: 10.1186/s12964-022-00916-1 (PMC9290307; doi:10.1186/s12964-022-00916-1)

**a**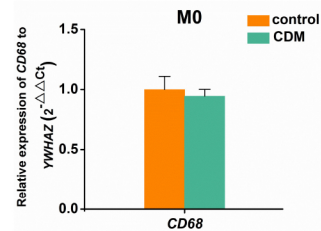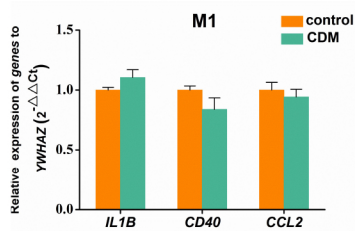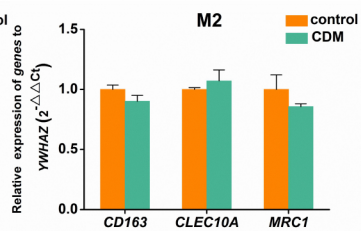**b**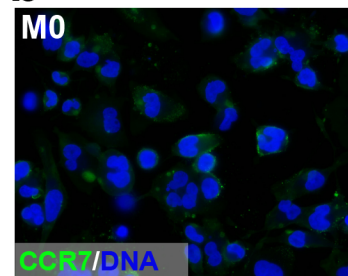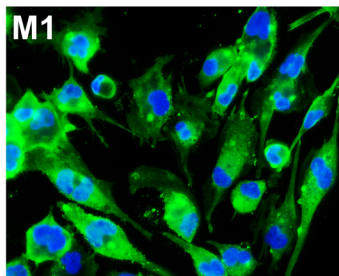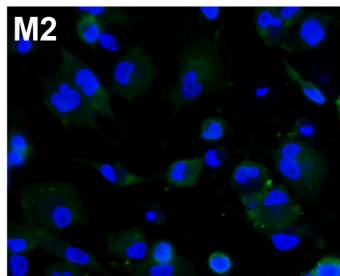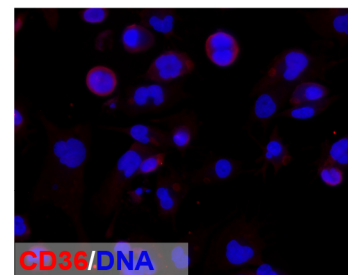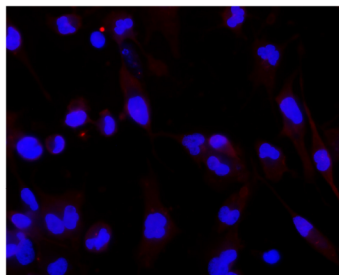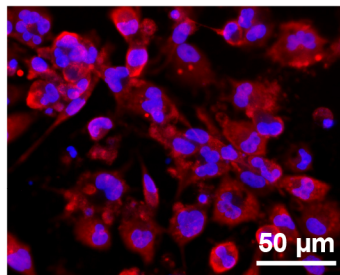

Supplement: Supplementary file 4 — Additional file 3. Supplemental Figure. [file 12964_2022_916_MOESM4_ESM.pdf]
